# Design and Implementation of a Dental English Learning Application Using Flutter

**DOI:** 10.7759/cureus.86481

**Published:** 2025-06-21

**Authors:** Yuta Otsuka, Tadahiro Higashinakao, Fumiko Nishio, Hiroshi Kono

**Affiliations:** 1 Department of Biomaterials Science, Graduate School of Medical and Dental Sciences, Kagoshima University, Kagoshima, JPN; 2 Department of Fixed Prosthodontics, Graduate School of Medical and Dental Sciences, Kagoshima University, Kagoshima, JPN

**Keywords:** dental education, educational tool, education app, english language, flutter

## Abstract

In recent years, as dental care becomes more internationalized, it is becoming increasingly important for dental students and dentists to learn English, especially dental terminology. However, there are still limited learning materials and environments specialized in such specialized vocabulary, and the development of efficient learning methods is a challenge. In this study, we developed a mobile application to support dental English vocabulary acquisition. Developed using the Flutter framework (Google, Mountain View, CA) and Dart language (Google), this app runs on a cross-platform compatible with iOS and Android, and is available through Firebase Hosting. The main features include a vocabulary list with 25 stages (20 words per stage), flash cards with voice read-aloud function using the speech application programming interface, and a multiple-choice quiz with random questions with no duplicate options. These features are designed to enable learners to efficiently memorize vocabulary from both a visual and auditory perspective.

A trial evaluation by the developer suggested that the app is suitable for short-term, repetitive learning and is useful for promoting vocabulary retention. In the future, we plan to improve the features by incorporating user feedback and conducting a formal evaluation study to verify the effectiveness of vocabulary acquisition.

## Introduction

In recent years, as opportunities for international exchange and information dissemination in dental care have increased, the importance of English proficiency required of dentists and dental students in Japan has increased. The ability to accurately understand and use specialized dental English vocabulary is essential for presentations at academic conferences, reading international papers, and dealing with foreign patients. However, university education and existing language education programs focus on general English, and there are few learning environments and materials for dental English that show translations of specialized Japanese and are specialized for dental terminology. In Japan, the use of mobile applications has become common for vocabulary learning support in university entrance exams and IELTS (International English Language Testing System) and TOEFL (Test of English as a Foreign Language) due to the spread of smartphones [1]. While general-purpose vocabulary apps such as Quizlet [2,3] and Anki [4,5] are flexible, they have issues in terms of the comprehensiveness of vocabulary in specific specialized fields and the accuracy of terminology. Anki has been shown in cohort studies to be effective in medical school education [6]. In addition, the user interface design and optimization of learning algorithms to promote memory retention are not uniform, and there are large individual differences in learning effectiveness [6].

In this study, we developed a mobile vocabulary learning application specialized for dental English vocabulary and report its design concept, functional configuration, technical implementation, and subjective evaluation by the developer. This app features flashcards and multiple-choice quizzes that emphasize the convenience and continuity of learners, and is designed to directly reflect the educational needs of the field. The app was developed using the Flutter framework (Google, Mountain View, CA) and Dart language (Google) [7], with the aim of providing a lightweight and highly responsive user experience while enabling cross-platform operation.

This paper discusses the background and purpose of the application development, details of the system design and implementation and initial evaluation results, and the direction of future applications and improvements.

## Materials and methods

Development policy and target users

This application was designed primarily for dental students and beginners aspiring to become dental medical professionals in Japan. The purpose is to provide efficient and continuous support for the acquisition of technical vocabulary, with emphasis on optimization for smartphones, especially for use while traveling or during free time. The interface is simple and intuitive, and consideration is given to allowing learners to repeat learning without hesitation.

Technology stack

Flutter was used for development and was implemented as a cross-platform application compatible with both iOS and Android platforms. English vocabulary data are structured in CSV format and classified into 25 stages (20 words per stage) using management numbers. Firebase Hosting [8] was used for application deployment and update management, and an environment that can be accessed directly from the web was established.

Functional configuration

The application is integrated with multiple functions to support vocabulary acquisition. First, the vocabulary list function presents specialized vocabulary in stages (25 stages, 20 words per stage) and provides a list with both English words and Japanese translations. Each stage is designed to be autonomous, so learners can proceed with their learning in any order.

The flash card function simultaneously displays the English and Japanese translations for each word, and a voice read-aloud function using Web Speech API (application programming interface) is implemented to assist memorization. This enables multisensory vocabulary learning using both visual and auditory pathways.

In addition, in quiz mode, multiple-choice questions are presented randomly, and the algorithm is designed to prevent overlapping of options. After answering, the correct answer is displayed immediately, allowing learners to immediately check their own level of understanding. Quiz questions and options are also read aloud by Web Speech API, enhancing vocabulary recognition through auditory stimulation.

## Results

**Figure 1:**
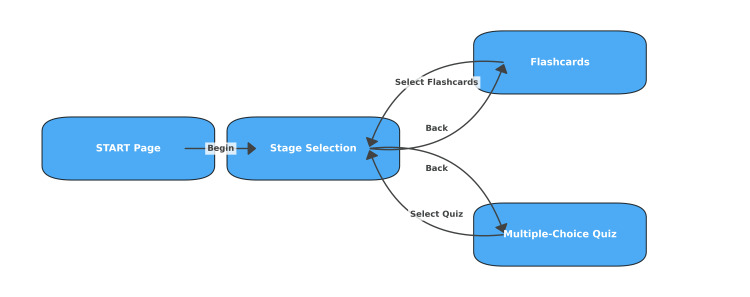
The scene configuration in DentEng application. The app consists of a start page, stage selection, flash card memorization page, and multiple-choice quiz. The app was developed by the authors.

This application has been released by the authors as a stable prototype as of April 2025 and has implemented several functions to support vocabulary learning. Figure 1 shows a page transition graph for the scene configuration. Figure 1 shows the scene configuration in the DentEng application. It consists of a start page, stage selection, flash card memorization page, and multiple-choice quiz.

This app is configured so that students can transition by tapping a button. The start page has a simple configuration that displays the title, a fun tooth character, a picture of a dentist, and the author's name in the lower right corner. In the stage selection, the vocabulary list consists of 25 stages (20 words per stage) and contains a total of 500 dental English words. Each word is accompanied by a Japanese translation and is designed to support systematic learning of technical terms. Table 1 shows a list of word examples.

**Table 1:** The list of English vocabulary in stage 1. A collection of basic English and Japanese terms related to teeth, which is a part of the translations in the app's list. The examples of words that are often used for learning dental English and for translations in the medical field.

Stage	English	Japanese
1	Gingiva	歯肉 (*shiniku*)
1	Caries	う蝕 (*ushoku*)
1	Enamel	エナメル質 (*enamelusitsu*)
1	Dentin	象牙質 (*zougeshitsu*)
1	Plaque	プラーク (*plaque*)
1	Calculus	歯石 (*siseki*)
1	Periodontium	歯周組織 (*sisyu sosiki*)
1	Pulp	歯髄 (*sizui*)
1	Cementum	セメント質 (*cement situ*)
1	Alveolar bone	歯槽骨 (*sisou kotsu*)
1	Oral mucosa	口腔粘膜 (*koukuu mennmaku*)
1	Saliva	唾液 (*daeki*)
1	Tooth	歯 (*ha*)
1	Jaw	顎 (*ago*)
1	Palate	口蓋 (*kougai*)
1	Tongue	舌 (*zetsu*)
1	Frenum	小帯 (*syoutai*)
1	Vestibule	前庭 (*zentei*)
1	Occlusion	咬合 (*kougou*)
1	Temporomandibular joint	顎関節 (*gakukansetsu*)

**Figure 2:**
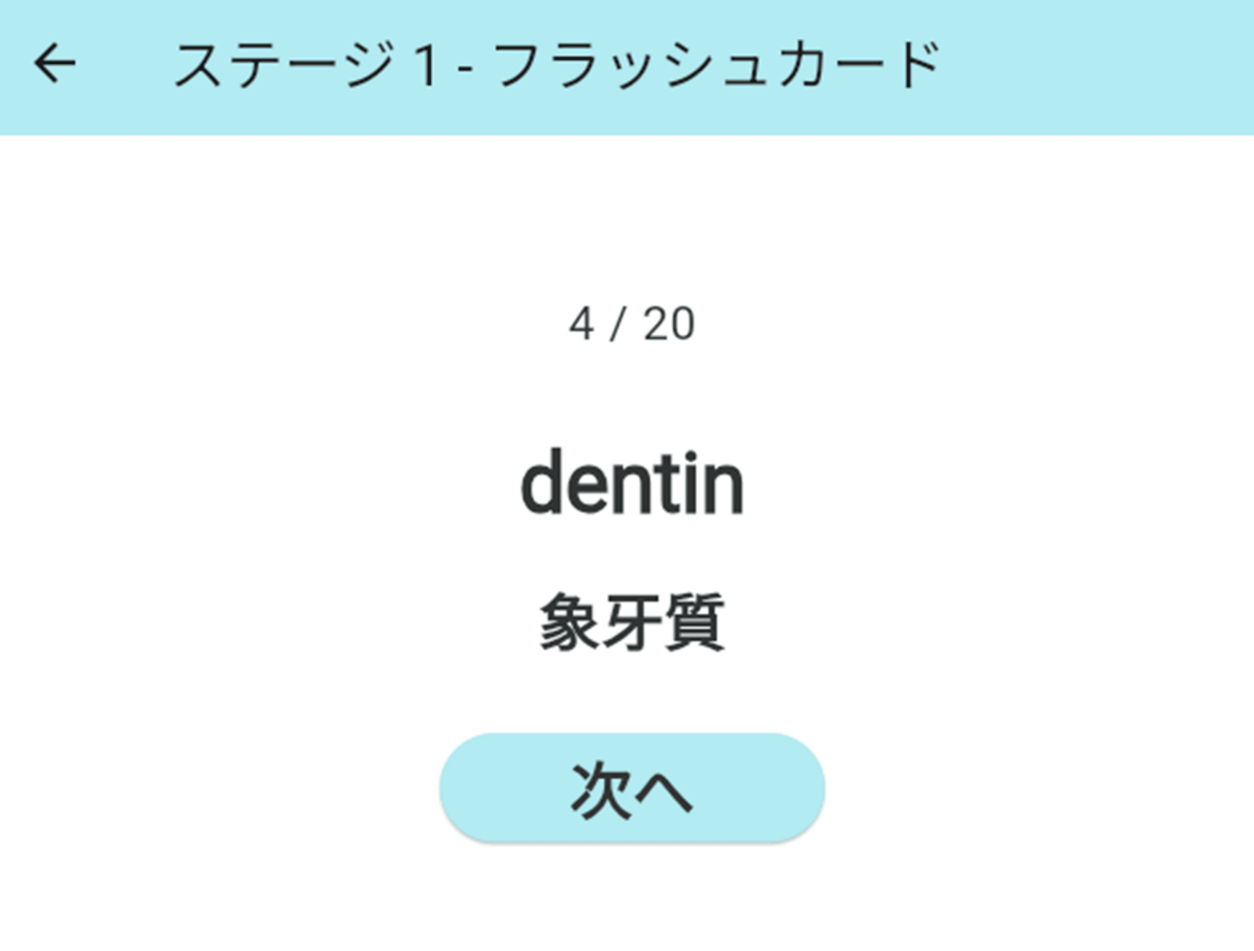
The image of the flash card. English and Japanese were shown in the monitor with pronounce audio. The word "Dentin" (象牙質) in English and Japanese is shown in the monitor with pronounce audio, flash card (フラッシュカード), stage 1 (ステージ1), and next button (次へ).

Figure 2 shows an image of the flash card function. In the flash card function, the English word and its Japanese translation are displayed simultaneously, reducing cognitive load by presenting visual information. In addition, the text-to-speech function uses the Web Speech API, which allows natural audio playback on the browser. This allows for multi-sensory vocabulary learning that combines vision and hearing. Many words are written in *katakana*, but we took into consideration the possibility that learners may not know the spelling.

**Figure 3:**
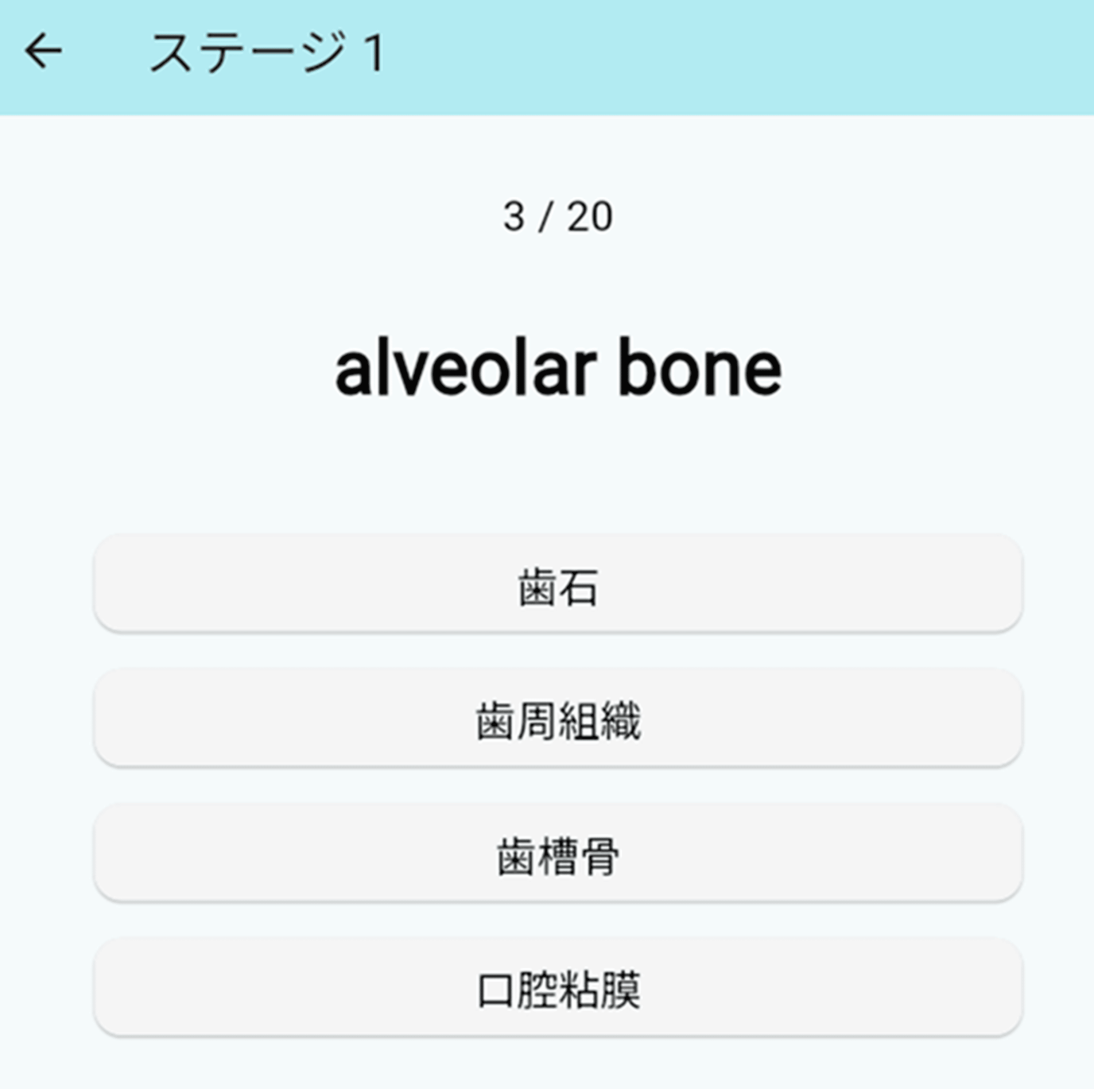
The image of the quiz screen in the app. The user can choose their answer from four Japanese options (calculus (歯石), periodontium (歯周組織), alveolar bone (歯槽骨), and oral mucosa (口腔粘膜)).

Figure 3 shows the quiz mode screen. Quiz mode is configured with four multiple-choice questions, and the algorithm is designed to randomly select questions so that no options overlap. After selecting an answer, immediate feedback is given on whether the answer is correct or not, promoting the correction of incorrect answers and memory retention. Both the questions and the options are read aloud by the Web Speech API, allowing learners to deepen their understanding of vocabulary through the multiple actions of "seeing," "listening," and "selecting."

This application can be accessed and used directly from mobile devices such as smartphones and tablets. The implemented prototype is publicly available at the following URL, and normal operation and responsiveness have been confirmed in major browsers (Chrome, Safari, etc.): https://denteng-62fd3.web.app/.

At present, no formal user survey that passed ethical review has been conducted, but through trial use by the developer himself on multiple mobile devices, the following usefulness was subjectively confirmed. First, the stage structure makes the learning progress clear, and the fact that vocabulary can be acquired while gaining a sense of gradual accomplishment was evaluated as effective for continuing learning. In addition, the flashcards have a voice read-out, which makes it possible to assist memory without relying solely on visual information. In quiz mode, the variety of options in the question format and the immediate feedback gave the impression that concentration was easily maintained.

Furthermore, the operability on smartphones was good, and it was confirmed that it is suitable for short-term learning and use while on the move. On the other hand, functions such as saving learning history and visualizing grades have not yet been implemented, and the introduction of a user tracking function is desired as a future improvement.

## Discussion

In this study, we developed a mobile-compatible learning application to support the efficient acquisition of dental vocabulary. This application integrates several features to reduce the burden on learners and encourage continuous learning, such as vocabulary organization by stage system, instant-display flash cards with translation, and confirmation learning by multiple-choice quizzes and instant feedback.

Vocabulary learning to date has relied on paper-based wordbooks and existing general-purpose vocabulary applications such as Anki, but this application, with its structure and software design specialized for specialized vocabulary, provides more direct support for learners in the dental field. In particular, the reduction of cognitive load by simultaneously displaying vocabulary and its meaning, and the design of feedback learning by instantaneous correct/incorrect judgment are thought to be useful for beginners.

In addition, the user interface structure optimized for use on mobile devices is suitable for short-term and repetitive use, and functions as an element to lower the hurdle of learning. The fact that learners can freely select stages and learn vocabulary in any order can also be evaluated as a design that supports an independent learning style.

From the perspective of learning consolidation, a design based on the immediacy and repetition of information presentation is thought to be effective in promoting memory consolidation. In particular, the flash card format that simultaneously displays the translation explicitly presents the connection between vocabulary and meaning, which is expected to smooth the transition to semantic memory. Furthermore, by using audio read-out in addition, input from multiple visual and auditory routes contributes to memory strengthening.

In addition, the immediate feedback function in the multiple-choice quiz is based on the hypothesis that memory consolidation based on errors and tests is efficient for memory consolidation [9], and is designed to correct incorrect answers and reinforce correct answers in real time [10]. In addition, the app is designed for short-term, high-frequency use, which may also contribute to long-term memory consolidation through distributed learning.

However, this study also has some limitations. First, a formal user evaluation and verification of the vocabulary acquisition effect requires an ethical review survey, and the subjective observation-based evaluation is limited to the developer's perspective. In addition, the current version does not support recording of learning progress, presentation of review history, or personalization based on incorrect answer trends, so there is room for further functional enhancement.

In the future, it is hoped that interactive functions such as progress management based on user behavior logs, automatic recording and review suggestions of incorrect vocabulary, and pronunciation checks will be introduced. In addition, by incorporating practical context such as the source and frequency of use of vocabulary and its application to clinical scenarios, it is possible to develop more practical vocabulary usage skills.

Developing educational apps using Flutter is also an opportunity for educators to increase their activities. The effects are not limited to the students who directly use it, but if it is widely used, it can have a wide range of educational effects. However, there is a possibility that an issue will arise regarding who will bear the server costs, and in fact, there are still some doubts about its use.

## Conclusions

In this study, we developed an application to support the learning of English technical vocabulary for dental medical students and reported on its functional design and usability. This application integrates various functions to support vocabulary acquisition for beginners, such as a 25-stage vocabulary list, flash cards with translations, multiple-choice quizzes with immediate feedback, and a text-to-speech function.

The user interface is optimized for mobile devices, and simple screen transitions that follow the learning flow are designed to promote continuous learning, providing a multisensory learning experience that utilizes both visual and auditory senses. At this point, the evaluation is limited to the subjective use evaluation, but it was suggested that a design that takes memory consolidation into account in the vocabulary presentation format and quiz structure may be effective. This application demonstrates its effectiveness as a method of vocabulary learning support in specialized fields, and we plan to develop it with an eye toward applications in other medical fields and multilingual support in the future.
